# "Will they just pack up and leave?" – attitudes and intended behaviour of hospital health care workers during an influenza pandemic

**DOI:** 10.1186/1472-6963-9-30

**Published:** 2009-02-13

**Authors:** Holly Seale, Julie Leask, Kieren Po, C Raina MacIntyre

**Affiliations:** 1 School of Public Health and Community Medicine, Faculty of Medicine, University of New South Wales, Kensington, New South Wales, Australia; 2 National Centre for Immunization Research and Surveillance of Vaccine Preventable Diseases (NCIRS), The Children's Hospital at Westmead, Discipline of Pediatrics and Child Health and School of Public Health, University of Sydney, Sydney, New South Wales, Australia; 3 Faculty of Medicine, University of Sydney, Sydney, NSW, Australia

## Abstract

**Background:**

There is a general consensus that another influenza pandemic is inevitable. Although health care workers (HCWs) are essential to the health system response, there are few studies exploring HCW attitudes to pandemic influenza. The aim of this study was to explore HCWs knowledge, attitudes and intended behaviour towards pandemic influenza.

**Methods:**

Cross-sectional investigation of a convenience sample of clinical and non-clinical HCWs from two tertiary-referral teaching hospitals in Sydney, Australia was conducted between June 4 and October 19, 2007. The self-administered questionnaire was distributed to hospital personal from 40 different wards and departments. The main outcome measures were intentions regarding work attendance and quarantine, antiviral use and perceived preparation.

**Results:**

Respondents were categorized into four main groups by occupation: Nursing (47.5%), Medical (26.0%), Allied (15.3%) and Ancillary (11.2%). Our study found that most HCWs perceived pandemic influenza to be very serious (80.9%, n = 873) but less than half were able to correctly define it (43.9%, n = 473). Only 24.8% of respondents believed their department to be prepared for a pandemic, but nonetheless most were willing to work during a pandemic if a patient or colleague had influenza. The main determinants of variation in our study were occupational factors, demographics and health beliefs. Non-clinical staff were significantly most likely to be unsure of their intentions (OR 1.43, p < 0.001). Only 42.5% (n = 459) of respondents considered that neuraminidase inhibitor antiviral medications (oseltamivir/zanamivir) would protect them against pandemic influenza, whereas 77.5% (n = 836) believed that vaccination would be of benefit.

**Conclusion:**

We identified two issues that could undermine the best of pandemic plans – the first, a low level of confidence in antivirals as an effective measure; secondly, that non-clinical workers are an overlooked group whose lack of knowledge and awareness could undermine pandemic plans. Other issues included a high level of confidence in dietary measures to protect against influenza, and a belief among ancillary workers that antibiotics would be protective. All health care worker strategies should include non clinical and ancillary staff to ensure adequate business continuity for hospitals. HCW education, psychosocial support and staff communication could improve knowledge of appropriate pandemic interventions and confidence in antivirals.

## Background

Since the start of the decade, endemicity and expanding outbreaks of avian influenza A/H5N1 have highlighted the threat of a future global influenza pandemic [1,2]. During the last year, transmission of avian influenza to humans has been reported in several countries. Up until now there has been little evidence that avian influenza is able to spread among humans, but if this were to happen, a pandemic may develop. Experts at WHO believe that "the world is now closer to another influenza pandemic than at any time since 1968" [3].

Estimates of the impact of a potential influenza pandemic range from 2 to 7.4 million deaths globally. In high income countries alone, models project a demand for 134–233 million outpatient visits and 1.5–5.2 million hospital admissions. Recent estimates from the UK suggest that up to half of the UK population could become infected (30 million people), with between 50,000 and 750,000 additional deaths as a result [4].

During a pandemic, health care workers (HCWs) will be essential to the health system response. Pandemic plans often specify that, in addition to patient care, HCWs will be involved in public health education, epidemiological surveillance, quarantine management, fever clinics, staging facility operation, and more [5,6]. Complicating this, however, are the various conflicting ethical and psychosocial issues relating to HCWs during an influenza pandemic: allocation and rationing of limited health resources, HCWs' professional versus family responsibilities, staff absenteeism and workforce issues, the risk to HCWs' personal safety, restrictions of personal liberties during quarantine, amongst others [7]. Therefore, an understanding of HCW knowledge, attitudes and behaviour is crucial in planning for an influenza pandemic. Addressing these issues is particularly important, as it has been argued that the threat of pandemic influenza may lead to aimless or unreasonable actions by health care workers [8]. Therefore the purpose of this study was to extend previous research by assessing the knowledge and intended behaviour during an influenza pandemic amongst clinical and non-clinical hospital staff.

## Methods

Between June 4 and October 19, 2007, we conducted a cross-sectional survey in two tertiary-referral teaching hospitals (one adult and one paediatric) to determine HCW's knowledge of pandemic influenza and behavioural intentions during that period.

### Survey

We developed an anonymous 2 page survey that assessed the following characteristics of participants: (1) Demographic characteristics, family situation, specialty; (2) Pandemic influenza knowledge and perceptions; (3) Intended behaviour in the event of a pandemic; (4) Intended compliance with public health measures; (5) Perception of pandemic influenza preparedness; and (6) Knowledge of infection control measures. Tick boxes were provided for responses to questions which were all closed.

The original version of the questionnaire was pilot tested, with five health care workers who were similar in their characteristics to the members of the study population, in order to ensure practicability, validity, and interpretation of answers. The questionnaire was revised before the distribution to the study sample, on the basis of the suggestions and comments obtained from the pilot study. Instrument revision included changes to questionnaire item wording and format only if there was near universal consensus on their meaning. The survey is available from the lead author upon request.

### Participants and Sampling

We contacted the head of each of the 40 wards or departments by either e-mail or letter to obtain permission to attend appropriate meetings to distribute surveys. These wards/departments were randomly selected from a list of all wards/departments in each of the hospitals. There were 27 wards from the Children's Hospital and 13 from the adult's hospital. A convenience sample of 1200 (15%) staff members was randomly selected to be surveyed from the 8000 staff members who work in the two hospitals. Only 894 staff members returned the survey.

Participants returned the survey to us immediately or by interoffice mail. We also met with Nurse Unit Managers from participating wards/departments, who agreed to distribute the survey amongst nurses and other staff. The surveys were returned in a central envelope in a secure location. The questionnaire was also available on the intranet, which was available to staff in the paediatric hospital only. Access to the electronic questionnaire depended on the hospital staff (from all four categories) having access to the hospital computer system.

Four groups were surveyed: Medical (Staff specialists, registrars, medical students etc), Nursing (registered nurses, nurse unit managers, and enrolled nurses etc), Allied Health Personnel (physiotherapists, occupational therapists, psychologists etc) and Ancillary staff (domestic services, administration, computer specialists etc). The latter group also included any staff member who was deemed to not have direct patient contact including academic staff and other public health professional staff.

To keep track of the departments who responded and to identify those who required follow-up, each ward/department was individually coded with an identification number, known only to the research investigators. To encourage participation amongst those wards/departments who failed to return the questionnaires, and in order to increase the response rate, emails were sent to the head of the department to ask them to encourage staff participation.

### Data analysis, Funding Support and Ethical Approval

The χ2 or Fisher exact test was used to assess the statistical significance of categorical variables. We considered results to be statistically significant with p < 0.05 via the Fisher's exact test. All analyses were performed using the OpenEpi 2.2 statistical package [9]. The survey was supported by the National Centre for Immunization Research and Surveillance, Children's Hospital at Westmead. Ethical approval was sought and granted from both of the hospitals.

## Results

A total of 1200 surveys were distributed (paper surveys) and 894 collected in the period of June 4 and October 19, 2007 (response rate: 74.5%). A further 185 questionnaires were submitted electronically from staff at the paediatric hospital, resulting in a total of 1079 completed questionnaires. We received 559 surveys (52%) from the paediatric and 520 surveys (48%) from the adult hospital. Respondents were categorized into four main groups by occupation: Nursing (47.5%), Medical (26.0%), Allied (15.3%) and Ancillary (11.2%). In the paediatric hospital, 28% (559/2000) of the staff members completed the survey. In the adult's hospital, 8.6% (520/6000) of the staff members completed the survey. Participant's occupational and demographic characteristics are summarized in Table 1.

**Table 1:** Demographic Characteristics of Participating Healthcare Workers (HCWs)

Characteristic	No. (%) of HCWs (n = 1079)
**Hospital**	
General	520 (48.1)
Pediatric	559 (51.8)
**Occupational cohort (three largest categories)**	
Nursing*	512 (47.5)
Registered Nurse	318
Clinical Nurse Supervisor	67
Nurse Unit Manager	27
Medical*	281 (26.0)
Staff Specialist	76
Registrar	64
Medical Student	65
Allied health*	165 (15.3)
Physiotherapist	36
Occupational Therapist	22
Social Work	11
Ancillary/hospital support*	121 (11.2)
Administration	72
Domestic Services	22
Catering	20
**Sex**	
Female	807 (74.8)
Male	245 (22.7)
Not specified	27 (2.50)
**Home/living arrangements**	
Live alone	108 (10.0)
Live in shared accommodation	267 (24.7)
Live with partner/spouse	132 (12.2)
Live with partner/spouse and children	446 (41.3)
Other	98 (9.10)
Not specified	28 (2.60)
**Age group (years)**	
18–30	338 (31.3)
31–40	280 (25.9)
41–50	247 (22.9)
51+	186 (17.2)
Not specified	28 (2.6)

* Nursing staff categories (Nurse Unit Manager, Clinical Nurse Co-ordinator, Clinical Nurse Supervisor, Registered Nurse, Enrolled Nurse, Student Nurse, Nurse Educator); Medical staff categories (Staff Specialist, Visiting Medical Officer, Registrar, Resident, Intern, Research Fellow, Medical Student); Allied Health staff category (Occupational Therapist, Physiotherapist, Dietician, Speech Therapist, Psychologist, Social Worker, Blood Collection, Laboratory Technician); Ancillary staff categories (Domestic services, IT, Administration, Security, Maintenance, Chaplain, Intake Officer, Finance).

Of 1079 respondents, 43.8% (n = 473) were able to correctly identify the meaning of "pandemic influenza", whilst 54.4% (n = 559) incorrectly labelled a pandemic as being either "a large outbreak of influenza in a given country or geographic area or yearly cases of influenza" (Table 2). 63.0% of ancillary staff was unable to correctly identify what a pandemic was. Medical staff was significantly more likely to select the correct definition than nurses. The majority of those surveyed considered that an influenza pandemic would be "very serious" if one were to occur. Groups significantly more likely to consider an influenza pandemic to be "very serious" were: front-line clinical staff (medical and nursing) (82.8%; OR 1.52, p < .01) and respondents who correctly defined pandemic influenza (88.1%; OR 2.45, p < .001).

**Table 2:** Knowledge and perceptions of pandemic influenza by HCW category

	HCW category Total, (%)				
	Medical (n = 281)	Nursing (n = 512)	Allied (n = 165)	Ancillary (n = 121)	Overall (N = 1079)

**What is pandemic influenza?**					
Yearly cases of the 'Flu'	2 (0.70)	12 (2.30)	3 (1.80)	11 (9.10)	28 (2.60)
A large outbreak in a given country/area	97 (34.5)	297 (58.0)	102 (62.6)	63 (52.1)	559 (51.8)
Influenza affecting birds	2 (0.70)	1 (0.20)	0	0	3 (0.30)
A disease which affects people in Asia	0	0	1 (0.60)	0	1 (0.10)
Global outbreak of a new influenza virus	180 (64.1)	192 (37.5)	54 (35.0)	44 (36.4)	473 (43.8)
Unsure	0	10 (2.00)	0	3 (2.50)	15 (1.40)
**How serious would a pandemic be if it occurred?**					
Not serious	0	2 (0.40)	0	0	2 (0.19)
Somewhat	33(11.7)	67 (13.1)	35 (21.5)	21 (17.4)	156 (14.5)
Very serious	244 (86.8)	413 (80.7)	125 (76.7)	91 (75.2)	873 (80.9)
Unsure	3 (1.10)	16 (3.10)	3 (1.80)	5 (4.10)	29 (2.69)
Not specified	1 (0.40)	14 (2.70)	0	4 (3.30)	19 (1.76)
**Is your ward/department prepared?**					
Yes	44 (15.7)	114 (22.3)	50 (30.7)	60 (49.6)	268 (24.8)
No	218 (77.6)	353 (68.9)	103 (63.2)	54 (44.6)	728 (67.5)
Not specified	19 (6.80)	45 (8.80)	10 (6.10)	7 (5.80)	83 (7.70)

When asked whether they consider their ward/department to be sufficiently prepared for an influenza pandemic, only 24.8% (n = 268) responded in the affirmative. Medical and nursing staff (19.9%; OR 0.4071, p < .001) and those who considered pandemic influenza to be very serious (23.4%; OR 0.6629, p < .01) were significantly less likely to consider their ward/department prepared, whilst ancillary and support staff were significantly more likely to consider their ward/department to be sufficiently prepared (49.5%; OR 3.54, p < .001).

In the event of an influenza pandemic, 83.3% (n = 899) of respondents indicated that they would present to work if a patient in their ward/department had an influenza-like illness, whilst 79.0% (n = 852) would present to work if a colleague had contracted pandemic influenza and 60.6% (n = 654) if a family member had an influenza-like illness. Most would not present to work if they themselves had symptoms consistent with influenza (81.2%, n = 876), including in the context of a severe staff shortage (53.4%, n = 576). Of the medical staff, 23.5% stated that they would attend work if they had symptoms during a severe staff shortage, however 48.0% would stay home if a family member was unwell. For nursing and allied health staff, 15.0% and 26.4% stated respectively that they would turn up to work if they had symptoms during a work shortage and 35% of respondents from both categories would not attend work if a family member was unwell. Non-clinical staff (ancillary/support) were significantly more likely to be unsure of their intentions (OR 1.43, p < .001). Factors significantly associated with work avoidance were: HCW category (nursing) and not correctly knowing what a pandemic was. Whereas, factors significantly associated with inappropriate work behaviour (such as turning up to work with an ILI) during a potential pandemic included age, HCW category (non-clinical), and perceived seriousness of pandemic influenza (Table 3).

**Table 3:** Results from univariate analysis of work practice in different scenarios

**Scenario**	**No.**	**OR**	**Lower**	**Upper**	**P value**
**Work avoidance**					
*Patient on ward has pandemic influenza*					
Female	45	1.45	0.69	3.13	NS
Have children	19	0.68	0.37	1.24	NS
Pandemic influenza "very serious"	43	0.77	0.39	1.53	NS
Medical/nursing	36	0.59	0.32	1.09	NS
*Colleague died of pandemic influenza*					
Female	57	1.11	0.60	2.06	NS
Have children	31	1.31	0.79	2.18	NS
Medical/nursing	46	0.50	0.30	0.83	<0.05
Incorrect knowledge	50	0.57	0.34	0.95	<0.05
**Inappropriate work practice**					
*Staff shortage/participant unwell*					
Age <40 years	158	2.66	1.85	3.83	<0.05
Incorrect knowledge	135	1.51	1.08	2.11	<0.05
Pandemic influenza "very serious"	164	0.58	0.39	0.86	<0.05
Pandemic influenza "somewhat serious"	45	1.75	1.14	2.69	<0.05
Medical/nursing	143	0.66	0.47	0.94	NS
Ancillary/support staff	31	1.52	0.92	2.48	<0.05

When asked whether they would comply with quarantine measures, in the event of an influenza pandemic, 45.0% (n = 486) of respondents intended to comply, a further 28.4% (n = 307) would comply but would be "very unhappy" about cooperating with the measures. Factors significantly affecting quarantine compliance and attitude (p < .05) were age <40 years, perceived seriousness of pandemic influenza and incorrect knowledge of pandemic influenza. Factors associated with participants feeling "unhappy" about quarantine included having children and being aged ≤40 years.

Staff indicated a high level of intended treatment adherence to any antiviral medications which may be provided to them in the event of an influenza pandemic, with 81.3% (n = 877) indicating an intention to take the course as instructed and only 6.8% (n = 73) indicating that they would divert the medications to family members. Factors affecting antiviral adherence and possible diversion to family members were: HCW category (ancillary/support), sex and age group. Those who were unhappy with quarantine were also significantly more likely to divert antivirals to their family (Table 4).

**Table 4:** Factors affecting compliance with public health measures during an influenza pandemic

**Scenario**	**No.**	**OR**	**Lower**	**Upper**	**P value**
**Will comply with quarantine measures**					
Age group <40 years	438	0.72	0.54	0.97	<0.05
Female	581	0.74	0.52	1.06	NS
Pandemic influenza "very serious"	655	1.58	1.13	2.22	<0.05
Pandemic influenza "somewhat serious"	101	0.62	0.43	0.91	<0.05
Incorrect knowledge	407	0.63	0.47	0.84	<0.05
Incorrect knowledge (only "large outbreak")	387	0.65	0.49	0.87	<0.05
**Very unhappy about quarantine**					
Have children	116	0.72	0.54	0.98	<0.05
Age group <40 years	190	1.54	1.13	2.09	<0.05
Incorrect knowledge	172	1.33	0.99	1.80	<0.05
Inappropriate work practice	62	1.48	0.99	2.21	<0.05
**Will take antivirals as directed**					
Medical/nursing	638	1.28	0.90	1.81	NS
Will comply with quarantine measures	665	1.93	1.38	2.70	<0.05
**Will give antivirals to family (all/some)**					
Female	45	0.49	0.29	0.84	<0.05
Have children	41	1.81	1.09	3.00	<0.05
Medical/nursing	40	0.46	0.27	0.77	<0.05
Ancillary/support staff	14	2.06	1.06	3.98	<0.05
Very unhappy about quarantine	25	1.76	0.95	3.26	<0.05

Only 42.5% (n = 459) of respondents considered that neuraminidase inhibitor antiviral medications (oseltamivir/zanamivir) would protect them against pandemic influenza, whereas 77.5% (n = 836) believed that vaccination would be of benefit. Medical staff (60.1%; OR 2.642, p < .001) were significantly more likely to consider neuraminidase inhibitors effective than any of the other groups surveyed, whereas ancillary staff were significantly more likely to believe that antibiotics would be effective for personal protection (18.2%; OR 1.954; p < .01). Nursing staff were significantly more likely to believe that eating well would be protective (65.8%; OR 2.338, p < .001). When asked what other interventions they considered would protect them from pandemic influenza, participants rated hand washing first (90.7%), followed by wearing gloves (69.6%), wearing a mask (81.8%), pandemic influenza vaccination (77.5%), and eating well (55.3%).

## Discussion

In the aftermath of SARS, many issues surrounding health care worker behaviour and professionalism have been discussed [10-14], however to the best of our knowledge, only a few studies have addressed these issues in relation to the anticipated influenza pandemic [15]. Our results suggest that most HCWs (83.3%) working in the two hospitals surveyed saw it as their professional obligation to treat sick patients and would continue working despite the potential risks. These results are in corroboration with previous studies from Hong Kong (84%) [16]. However, they differ from other research in Australia (67%) [17], and from the United States (50%) [18]. The differences may relate to non standard survey questions or a factor of time, with knowledge and intentions changing as exposure to information about PI increases. Suggested reasons for this include the fact that HCWs consider it unethical to abandon their professional responsibilities in order to protect themselves or their families [14,19].

When participants were asked whether they consider their ward/department to be sufficiently prepared for an influenza pandemic, only 24.8% responded in the affirmative. This high lack of confidence in the department's preparation may actually stem from a real lack of preparation by the hospital or department, or may just be a result of HCWs being unaware of any planning which has been conducted. Further studies would need to be conducted to ascertain which of these two options the most likely reason is.

While many health care workers will willingly attend work during an infectious diseases emergency, history provides many stories of physicians who have avoided responsibility for treating patients [20]. The appearance of an exotic, highly virulent disease, challenges HCWs to question their interpretation of the duty of care, in particular, its limits. This challenge was apparent both in the HIV/AIDS epidemics of the 1980s, where fear about contact with infected patients among some clinicians challenged their responsibilities to these patients and secondly in the 2003 SARs outbreaks [21-24]. There were several reports during the SARs outbreak that HCWs in Hong Kong and Toronto either avoided the physical examination of sick patients or refused to work altogether on the grounds that they presented too great a risk. In China, at the height of the SARs epidemic, at least one hospital had difficulty maintaining services due to absenteeism some of which was driven by fear of getting sick [25]. A recent survey assessing the willingness and ability of HCWs to report to duty during catastrophic disaster in New York City, found that although more than 80% were willing and/or able to report to work for mass casualty or environmental disaster, only 57% to 68% would be willing to report to work during a severe acute respiratory syndrome (SARS) or smallpox outbreak [12].

Fears for personal safety and family responsibilities are commonly the main issues underlying possibly absenteeism during a pandemic – in our study the rate of absenteeism doubled in the scenario of a family member being infected. Our finding was also echoed in the New York survey, where fears and concern for the family and for themselves were the most frequently stated reasons for not being willing to report to work [12]. Whilst in a second study, the authors reported that almost one third of the HCWs they surveyed either strongly agreed or agreed that it was professionally acceptable for HCWs to abandon their workplace during a pandemic in order to protect themselves and their families [19].

It is interesting to note that willingness to work varied considerably according to the individual's knowledge and their job classification. Medical and nursing staff was significantly more likely to report to work, whereas ancillary staff was unsure of their intentions during this period. A recent study by Ehrenstein et al, reported a similar finding in that the rate of administrators not willing to accept personal risk was approximately twice as high as the rate of other staff [18]. This difference may correlate with a perception of the importance of one's role in the hospital during the response. Whilst it's important to encourage all categories of staff members to turn up for work – it must be done in an appropriate manner. Inappropriate working behaviours were identified when participants were asked to nominate their potential response in a given situation. For example, when asked whether they would attend work during a staff shortage, if they had symptoms consistent with pandemic influenza, 24% of medical staff and 26% of ancillary staff said yes. In a second scenario, participants were asked whether they would attend work if a close family member was diagnosed. This time over half of the participants stated that they would. Whilst this may not be detrimental in light of antiviral and vaccine availability, in the early stages of a pandemic, this behaviour may be linked to the spread of the disease.

Quarantine is a key public health measure in pandemic influenza plans. It is also one of the oldest methods of controlling communicable disease outbreaks. Australia's pandemic influenza plans, amongst others, emphasize the use of quarantine measures (home quarantine for up to one week) in combination with social distancing measures and antiviral medications. Our study defined quarantine as involving: (i) being forbidden to use public transport, (ii) being allowed only to travel directly from home to work and back, (iii) being isolated from other family members in their home (as was enforced in Canada during the 2003 SARS outbreak) [26]. We found that most HCWs would comply with such quarantine measures (and adhere to antiviral medications), however, a large proportion of those surveyed stated that they would be very unhappy about it, which could ultimately affect compliance with the measures. Helping people to understand the reason for various protocols might increase their belief in their effectiveness and thus, their compliance. The use of education was previously examined in a post SARS article which examined a cohort of persons quarantined during the 2003 SARS outbreak in Canada [26]. The authors found that compliance could have been improved by providing enhanced education and support. The authors also felt that this could have also reduced the psychological distress in the quarantined adults.

In a study by Shiao et al, factors related to nurse's consideration of leaving their jobs during the SARS outbreak in Taiwan were examined [26]. The authors found that the main predictors were short tenure, increased work stress, perceived risk of fatality from SARS, and affected social relationships. On the contrary, belief in the effectiveness of personal protective equipment (PPE) was not an important predictive factor for nurse's consideration of leaving their job. This was because most nurses surveyed believed that protective measures at work were generally effective. While we cannot ascertain from our study whether there is a link between PPE effectiveness and work attendance, we can make postulations about the confidence the staff have in the different protective measures. For example, when respondents were asked what will protect you from getting pandemic influenza, the most common response was washing hands, wearing masks and vaccination. However, when it came to antiviral use, more respondents stated that "eating well" would offer better personal protection then the anti-viral drugs oseltamivir ("Tamiflu") or zanamvir ("Relenza") against pandemic influenza.

There are a number of limitations to this study that need to be discussed. It is important to recognize that the generalisability of our results may have been affected by the limitations inherent to any voluntary questionnaire-based cross sectional study. Given the design of the study, we relied on a convenience sample of 1200 staff members from the total number (8000) of hospital staff in both hospitals. From this number, only 894 actually returned the survey. As responses were voluntary, there may have been responder bias in the sample. We were unable to compare the demographics of the respondents versus non-respondents to examine how representative the sample actually was. This information was not available to the study authors. We can only rely on the fact that we obtained a 13.5% sample from the two hospitals. This sample is considerable large for such a study. Another limitation relates to the online response rate. The online invitation to complete the survey electronically was seen by an unknown number of staff at the paediatric hospital leading to a lack of denominator data about the 185 completing it. Nevertheless, the paper-based survey was comprehensive, yielding a large sample and acceptable response rate.

There may also be limitations with generalisability since we included only two hospitals in a single Australian city. It is also unknown as to whether responses given to the hypothetical situations posed in a questionnaire accurately reflect real-world responses of the respondents in the event of an actual influenza pandemic.

Despite these issues, the large sample size of our study, broad spectrum of HCWs represented, and representative age/gender demographics provide a general indication of what responses to a pandemic may occur and provide information on differences between health care worker groups. In addition, much research focusing on behavioural intentions indicates the potential for these to be reasonable in predicting actual behaviour [27].

## Conclusion

It is apparent from our findings that there are several issues that must be addressed as part of health system preparedness for a coming influenza pandemic: HCWs should be provided with appropriate targeted education and within this ancillary staff need to be included, the psychosocial needs and concerns of HCWs should be addressed in pandemic planning, communication regarding health resources/planning should be improved to develop staff confidence, and consideration could be given to families of HCWs also receiving priority access to the National Medical Stockpile. In the face of a pandemic influenza threat, health department employee's unwillingness to report to duty may pose a threat to the nation's emergency response infrastructure. This must include all classifications of staff which are fundamental to the hospital's operation. We feel that addressing these issues of importance to HCWs is essential to ensure an effective and appropriate health system response.

## Competing interests

The authors declare that they have no competing interests.

## Authors' contributions

HS participated in the design of the study and survey, undertook the distribution and collection, performed the analysis and drafted the manuscript. JL participated in the design of the study and survey and reviewed the manuscript. KP helped perform the statistical analysis and drafted the manuscript. CRM participated in its design and coordination and helped to draft the manuscript. All authors read and approved the final manuscript.

## Pre-publication history

The pre-publication history for this paper can be accessed here:


